# Educational Module Intervention for Radiographers to Reduce Repetition Rate of Routine Digital Chest Radiography in Makkah Region of Saudi Arabia Tertiary Hospitals: Protocol of a Quasi-Experimental Study

**DOI:** 10.2196/resprot.8007

**Published:** 2017-09-26

**Authors:** Abdullah A Almalki, Rosliza Abdul Manaf, Muhamad Hanafiah Juni, Hayati Kadir Shahar, Noramaliza Mohd Noor, Abdelsafi Gabbad

**Affiliations:** ^1^ Department of Community Health Faculty of Medicine and Health Sciences Universiti Putra Malaysia Selangor Malaysia; ^2^ Department of Imaging Faculty of Medicine and Health Science Universiti Putra Malaysia Selangor Malaysia; ^3^ Department of Epidemiology Collage of Health Science Al-leeth-Makkah Saudi Arabia

**Keywords:** repetition rate, intervention, radiographer, quasi-experimental, information motivation behavioral skills model

## Abstract

**Background:**

Repetition of an image is a critical event in any radiology department. When the repetition rate of routine digital chest radiographs is high, radiation exposure of staff and patients is increased. In addition, repetition consumes the equipment’s life span, thus affecting the annual budget of the department.

**Objective:**

The aim of this study is to determine the impact of a printed educational module on reducing the repetition rate of routine digital chest radiography among radiographers in Makkah Region tertiary hospitals.

**Methods:**

A quasi-experimental time series with a control group will be conducted in Makkah Region tertiary hospitals for 8 months starting in the second quarter of 2017. Four hospitals out of 5 in the region will be selected; 2 of them will be selected as the control group and the other 2 as the intervention group. Stratification and a simple random sampling technique will be used to sample 56 radiographers in each group. Pre- and postintervention assessments will be conducted to determine the radiographer knowledge, motivation, and skills and repetition rate of chest radiographs. Radiographs of the chest performed by sampled radiographers in the selected hospitals will be collected for 2 weeks before and after the intervention. A piloted questionnaire will be distributed and collected by a researcher in both groups. One-way multivariate analysis of variance and 2-way repeated multivariate analysis of variance will be used to analyze the data.

**Results:**

It is expected that the repetition rate in the intervention group will decline after implementing the intervention and the change will be statistically significant (*P*<.05). Furthermore, it is expected that the knowledge, motivation, and skill levels in the intervention group will increase significantly among radiographers after implementation of the intervention (*P*<.05). Meanwhile, knowledge, motivation, and skills in the control group will not change.

**Conclusions:**

A quasi-experimental time series with a control will be conducted to investigate the effect of printed educational material in reducing the repetition rate of routine digital chest radiographs among radiographers in tertiary hospitals in the Makkah Region of Saudi Arabia.

## Introduction

Good quality images in routine radiography should provide an adequate picture of the body’s anatomy. Failure to obtain a good quality image requires the radiograph to be repeated. According to Foos et al [1], the term “repetition” refers to redoing a radiograph of a patient that was deemed clinically unacceptable. Repetition of an image is a critical event in radiology. It is recommended that the repetition rate should not exceed 5% [2-7]. The Diagnostic Imaging Quality Assurance Committee recommends that the repetition of radiographs should not exceed 5% to 7% [8]. The American Association of Physicists in Medicine recommends keeping the repetition rate below 6%, and when it increases to 10%, corrective action should be conducted [9]. The Australian College of Radiologists recommends an acceptable repetition rate of 2% and not more than 5% [10].

A study by Khafaji and Hagi [11] reported high repetition rates of radiography in Saudi hospitals, averaging 14.9%, which is higher than the international standard. Another study reported the repetition rate in 3 Ministry of Health hospitals ranged from 7.4% to 9.7%. The same study revealed that chest radiographs have higher repetition rates compared to other radiological procedures [12]. Related to that, it was revealed that radiographer error is one of the factors that strongly contribute to the issue of the repetition [13,14].

The production of high-quality images is based on radiographer practices. According to the World Health Organization, practice is influenced by the level of knowledge, motivation, and skills [15]. A study conducted in Saudi Arabia by Alsharif et al [16] showed that there is poor knowledge among radiographers in identifying image error. Another study conducted in Saudi Arabia by Ahmed et al [17] revealed that there is variation in radiographer knowledge of radiation protection, with 58% of radiographers indicating poor knowledge. Additionally, it was revealed that the motivation level of radiographers is low and this affects production of high-quality images [18]. Lack of knowledge and motivation dramatically influence skill level. It has been exhibited that the increase in repetition rate is due to deficiencies in radiographer skills [19]. Radiographers with high skills tend to avoid errors in the imaging process. Skills include the ability to communicate properly with the patient and handling the equipment accurately.

Repeated radiographs have financial and health implications, especially because of increased exposure to radiation for both staff and patients [12]. Khafaji and Hagi [11] and Khoshinani and Heidari [20] added that a high repetition rate in radiography consumes the digital equipment’s lifetime by 2 months each year. This increases both staff workload and waiting time for the patient in addition to affecting the achievement of the organization’s vision.

According to Almalki et al [21], most interventions conducted in previous studies are technical in nature. Despite that, those intervention studies show a positive impact on the repetition rate of digital radiography. However, radiographers were not included in the studies despite them being significant factors in the repetition rate.

In general, the aim of this study is to develop, implement, and evaluate the impact of printed educational material in reducing the repetition rate of routine digital chest radiography among radiographers in Makkah Region tertiary hospitals.

## Methods

### Study Design

The design of this study is basically a quasi-experimental time series with a control group. This design was chosen because the intervention was recommended by other researchers [22,23]. A quasi-experimental study is the only design that could be applied in this study. The difficulty of randomizing by location and subject and the small number of the population make the quasi-experimental design suitable in this study [24]. In addition, there is difficulty in randomizing by subject to avoid potential contamination. Location is also a factor, since no 2 hospitals are similar.

**Figure 1:**
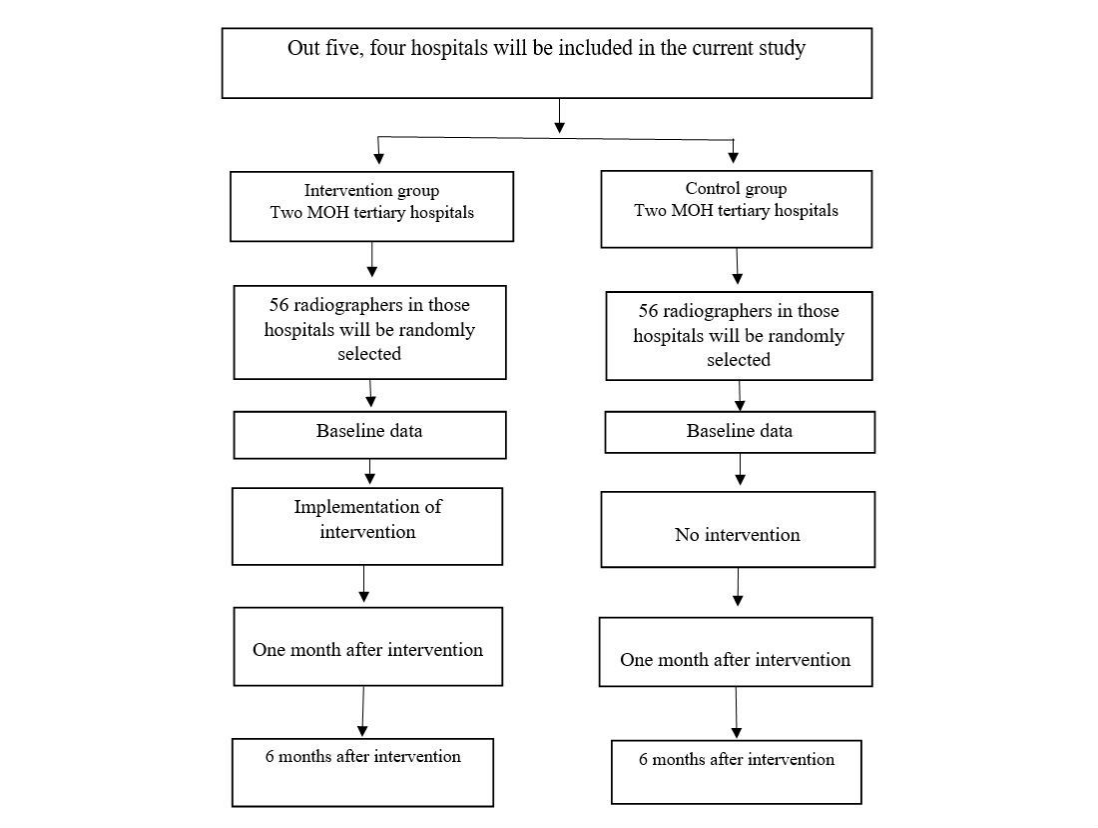
Study flow.

The Makkah Region of Saudi Arabia was selected for the study because the problem has been ignored in the area and the repetition rate has not been periodically measured there [25,26]. Out of 5 hospitals, 4 will be selected to be in the study since randomization is not required in this type of study [27]. The hospitals involved are under the direction of Makkah Region health affairs. Two will be chosen as an intervention group and 2 as a control group. Preintervention assessment will be conducted in both groups at the same time during the second quarter of 2017. The intervention will be distributed to radiographers in the intervention group, and after 1 month, an assessment will be conducted. Six months after the implementation of intervention, a second postintervention assessment will be conducted. Figure 1 demonstrates the study flow.

### Selection Criteria

The inclusion and exclusion criteria are based on the job description of a radiographer, since there are several tasks in routine radiography in the radiology department. Only radiographers who perform routine digital chest radiography will be included. Clerk radiographers, administrative radiographers of routine digital radiography, radiographers who are on long annual leave, and radiographers who are pregnant will be excluded. Non-Saudi radiographers will be excluded as well.

### Recruitments

#### Sampling Methods

The method employed to sample radiographers is stratification followed by simple random sampling. The list of radiographers will be obtained from the radiographer in charge. After that, the inclusion and exclusion criteria will be applied to radiographers derived from the list. Then, radiographers will be stratified by gender, with male respondents representing 70% of the sample and female respondents forming 30%. After that, a software number generator will be used to select the targeted sample.

#### Matching

Matching has been used in research since the beginning of the 19th century [28]. Exact matching is the method that will be employed in this study. Stuart and Rubin [29] recommended the selection of the most common covariate that has an effect on the outcome in order to make matching possible. According to Loman [30], exact matching can be employed for up to 5 variables. Based on these principles, variables that underwent matching include gender, experience, education level, training, and the type of university from which a radiographer graduated.

Respondent characteristics will be obtained from the radiographers in charge in the control group. First, a sample from the intervention group will be randomly selected using a software number generator. Since the list of radiographers in the control group and the characteristics have already been obtained, exact matching can be performed. This method will help in making the groups comparable and similar in terms of confounder distribution.

### Sample Size

**Figure 2:**
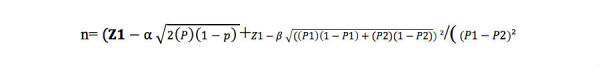
Formula of sample size estimation to test a hypothesis of proportion of 2 populations.

**Figure 3:**
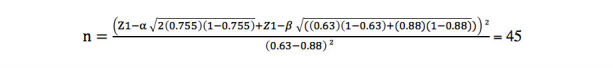
Sample size of the secondary outcomes.

We will use the formula by Lemeshow et al [31] to estimate the minimum sample size required in intervention studies and to test a hypothesis of proportion of 2 population problems in terms of the radiographers sample size (see Figure 2), where *p* 1 and *p* 2 were obtained from a study by Moreira [32] to estimate the sample size of radiographers in each group and Z1–α=1.96, Z1–β=0.842, *p* 1 is 0.63, *p* 2 is 0.88, and *p*^–^=0.755. Figure 3 displays the sample size estimation of the secondary outcome variables.

According to Sullivan [33], the attrition rate (dropout) can be calculated by the formula (desired sample size)/(percent retained). Hence, for this study, a sample size of 56 for each group was targeted.

**Figure 4:**
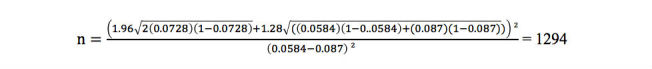
Sample size of the primary outcome.

In terms of the repetition rate of routine digital chest radiographs sample size, the same formula was used, where *p* 1 and *p* 1 were obtained from the study of Zhang and Chu [34] to estimate the sample size of routine digital chest radiography in each group and Z1–α=1.96, Z1–β=0.842, *p* 1 is 0.0584, *p* 2 is 0.087, and *p*^–^=0.0728. Figure 4 displays the sample size estimation of the primary outcome variable.

According to Sullivan [33], the attrition rate that may occur due to any loss of chest images can be calculated by the formula (desired sample size)/(percent retained). The sample size of routine digital chest radiographs is 1618 for each group, and this number is expected to be reached within 2 weeks. Two weeks’ time is similar to that used in the study conducted by Ahmed and Suliman [35].

### Instruments

A questionnaire developed by the researcher based on the information motivation behavioral skills model is one of the instruments that will be used to evaluate the level of radiographer knowledge about imaging, as well as the motivation and skills. It consists of close-ended questions and is divided into 2 sections: demographic data of the radiographer and domain of the radiographer’s knowledge, motivation, and skills. Radiographers are expected to spend 5 minutes completing the questionnaire.

A check list was recommended and used in several studies around the world to measure the repetition rate of routine digital chest radiographs [8]. It is an international instrument. It contains radiographer demographic data, number of radiographs performed by radiographer, number of repeated radiographs, and causes of repetition. It is completed by the researcher in order to obtain accurate results and overcome biases, using actual numbers. Therefore, its reliability does not need to be checked. Furthermore, studies conducted by Al-Malki et al [12] and Khafaji and Hagi [11] in Saudi Arabia used the same instrument. This means that the check list used in this study is valid.

In order to achieve accurate and precise results, the validity and readability of the questionnaire will be evaluated. Face validity will be ensured by an expert currently practicing to ensure the veracity of the meaning, wording, and sequences. Content validity will be ensured by lecturers working in the university to ensure clarity, representation, and comprehensiveness. Furthermore, factor analysis will be conducted to ensure a structural correlation between variables and factors on the instruments. Finally, reliability through the Cronbach coefficient alpha will be conducted to ensure internal consistency.

### Intervention

Piloted intervention will be used in this study. The intervention is in the form of printed educational material distributed to routine digital radiographers in the departments of intervention hospitals based on a specific module developed for the purpose of the study. The intervention module was developed from previous studies [36-39]. The education material was developed based on the information motivation behavioral skills model. This model has 4 constructs: information, motivation, skills, and behavioral change. This model was selected because it was recommended by another researcher to study the effect of self-efficacy, attitude, and knowledge on repetition. The intervention component comprises 3 sections. The first section touches on the background of the repetition issue and the importance of producing high-quality chest images. The second section encompasses the motivation issue of repetition and dose of radiation. The third section includes important skills that should be performed by a radiographer to reduce the repetition rate of chest images. Furthermore, the educational material discusses the issue of repeated radiography and the definition, repetition rate, international standard, causes of repetition, and the burden of repeated radiography to radiographers, patients, clinicians, and the organization. Anatomical parts which should be included in chest radiography will also be included in the education material.

### Outcome Measure

#### Primary Outcome

The primary outcome of this research is the repetition rate of routine digital chest radiographs. It is the change of the behavior based on the information motivation behavioral skills model.

#### Secondary Outcome

The secondary outcome in this study is knowledge, motivation, and skills of radiographers. Based on the information motivation behavioral skills model, there are direct and indirect correlations between knowledge and behavioral change. There are also direct and indirect correlations between motivation and behavior change. Meanwhile, behavioral skills have a direct correlation with behavioral change.

### Statistical Analysis

Data analysis in this study is divided into 2 parts: descriptive and inferential. Descriptive data will be calculated in order to compute the central tendency and dispersion to add valuable statistical information to the study. Inferential data analysis will be used to meet a specific objective. Chi-square, 1-way multivariate analysis of variance, 2-way repeated measure multivariate analysis of variance, and multivariate analysis of covariance are the statistical methods that will be used to test the hypothesis. *Cochran Q test* will be also employed to assess the difference in proportion. The level of significance will be set at α=0.05, and all testing of hypotheses will be conducted using 2-sided tailed hypotheses. The statistical program used is SPSS version 22 (IBM Corp).

### Ethics Approval and Registration

Approval from the ethics committee of the faculty of Medicine and Health Sciences of the University Putra Malaysia was obtained (reference number EXP16 P160). Approval to conduct the study was also obtained from the Ministry of Health (reference number H-02-J002). Approvals from Makkah health affairs and the hospitals that are under study were also obtained. In addition, radiographers who will be involved in the study will sign a consent form.

## Results

The researchers expect that the repetition rate and the radiographer knowledge, motivation, and skills in both the control and intervention groups before intervention are statistically not significant (*P*>.05). It is expected that a high repetition rate with a low level of knowledge, motivation, and skills in both groups will be found in the baseline data. We predict that after implementation of the educational material in intervention hospitals, the knowledge, motivation, and skills of radiographers will increase and the repetition rate will *reduce* (*P*<.05), but we do not expect the repetition rate, knowledge, motivation, and skills to change in the control group (*P*>.05). It is expected that the intervention will be effective to change the behavior and reduce the repetition rate of routine digital chest radiography (*P*<.05). The results are expected to be published in 2018.

## Discussion

### Summary

This quasi-experimental time series with control group aims to investigate the effect of printed educational material on radiographer knowledge, motivation, and skills and the radiography repetition rate.

Educating radiographers helps reduce the dose of radiation exposure on patients, decreases waiting time, and increases patient satisfaction. A reduction in the repetition rate decreases the dose of radiation and reduces the workload. This intervention is significant to the organization as it reduces the burden of equipment consumption and cost as well as assists the organization in achieving its vision and goals. In addition, implementing an educational program that focuses on reducing the repetition rate of radiographs has been highly recommended [12,40,41].

To our knowledge, this is the first study that combines 4 outcome variables—knowledge, motivation, skills, and the repetition rate of routine digital chest radiography—and aims to investigate the effect of using printed educational material on the repetition rate of routine digital chest radiography. Furthermore and based on our knowledge, this is the first study that analyzes the repetition rate among radiographers.

The intervention module will be made available in both English and Arabic languages, and participants can choose their preferred language to complete the sessions. The intervention program was designed to be as brief as possible to increase readability. The printed educational material was selected because of the difficulty of assembling radiographers from different cities in one place at one time. There is a need to overcome the issue of bias to increase the credibility of the study. The quasi-experimental design is one of the strongest designs for this particular research.

### Limitations

There are some limitations to this study beginning with the research design. The threat of internal validity mostly reduces the inference of causality due to the lack of randomization. However, the researcher will make the groups comparable and similar by using the exact matching technique. Another limitation is that the result cannot be generalized to all of the hospitals in the Makkah Region due to differences in hospital equipment, which may be conventional, computed, or direct forms of radiography. These modalities are totally different than the others, but the result can be generalized on tertiary hospitals in the region.

The printed education intervention could serve as a new modality to manage the critical event of repetition among radiographers. The study aims to provide better recognition and management of the repetition rate of routine digital radiography through increasing knowledge, motivation, and skills. It also aims to educate and create awareness of the problem of repetition in radiography. There is a need to develop simple, brief, and effective interventions tailored to the needs of the radiology department to reduce the burden of repetition among radiographers.

### Conclusion

To our knowledge, this study will be the first quasi-experimental time series study with a control group using a printed educational material intervention program for radiographers to investigate the repetition rate in chest radiography and radiographer knowledge, motivation, and skills. The results from this study will determine the effectiveness of the intervention in managing and decreasing the repetition rate of routine digital radiography among radiographers. If proven to be effective, the intervention can better serve the organization by assisting decision making in the radiology department to manage and reduce the burden caused by repetition.
